# Composition and random elimination of paternal chromosomes in a large population of wheat × barley (*Triticum aestivum* L. × *Hordeum vulgare* L.) hybrids

**DOI:** 10.1007/s00299-019-02405-1

**Published:** 2019-04-06

**Authors:** Dávid Polgári, Edit Mihók, László Sági

**Affiliations:** 1 Centre for Agricultural Research, Hungarian Academy of Sciences, Martonvásár, 2462 Hungary; 2 Szent István University, Gödöllő, 2100 Hungary

**Keywords:** Cereals, Chromosome marker, GISH, Ultrawide cross, Uniparental genome elimination

## Abstract

**Key message:**

Statistical analysis of the chromosomal composition in a population of 210 primary plants regenerated from two intergeneric wheat–barley cross combinations revealed the random nature of uniparental elimination for barley chromosomes.

**Abstract:**

Uniparental chromosome elimination is a common process in interspecific and intergeneric cereal hybrids. To characterize the frequency of paternal chromosomes, a population of 218 independent green plants was generated from two wheat (♀) × barley (♂) cross combinations via embryo rescue. The chromosomal composition of 210 primary plants was analyzed with chromosome-specific DNA markers representing all seven barley chromosomes. The analysis revealed an equal proportion of haploid and full hybrids (20.5% and 19.5%, respectively), while the rest of the population contained hypoploids (partial hybrids) with no preference for any possible numbers (one to six) of barley chromosome additions. Contrary to the previous reports, there was no statistical bias or preferential elimination for any individual barley chromosome (1H–7H) in this population. The reasons for the apparent contradiction and the implications of the above findings for cereal breeding are discussed.

**Electronic supplementary material:**

The online version of this article (10.1007/s00299-019-02405-1) contains supplementary material, which is available to authorized users.

## Introduction

Complete or partial uniparental genome (or chromosome) elimination during initial mitotic divisions in embryos of ultrawide (interspecific and intergeneric) sexual hybrids is a general phenomenon in the plant kingdom (Ishii et al. 2016). It represents a key element in the protection of genome integrity from “genome shock” (McClintock 1984) as a result of hybridization between unusually distant partners. Uniparental chromosome elimination is particularly well described for cereal plant species, where it is primarily, though not exclusively, confined to the paternal genome donor (Houben et al. 2011; Zhao et al. 2013).

The precise mechanism of chromosome elimination is complex and appears to be species-specific. Besides the differential rates of cell cycle in the parental genomes (Bennett et al. 1976), several alternative mechanisms have been proposed such as a markedly different (or lack of) attachment of the two parental chromosome sets to microtubuli during early mitotic divisions in wheat × maize zygotes (Mochida et al. 2004). In other cases, chromosome elimination during the embryogenesis of cultivated barley (*Hordeum vulgare*) × *Hordeum bulbosum* hybrids is associated with the formation of micronuclei, abnormally condensed chromatin, and chromosome fragments (Gernand et al. 2006). These rearranged chromosomes and micronuclei are derived from the breakage of bridges and retention of acentric fragments in anaphase, respectively. Thus, chromosome elimination is not always due to malfunction of the kinetochores binding to the microtubuli but also to the failure of the sister chromatids to segregate at anaphase (Ishii et al. 2010). More recently, uniparental centromere inactivation was proposed as the cause of paternal chromosome elimination in wheat or barley × *H. bulbosum* hybrids (Sanei et al. 2011). Clearly, different mechanisms of chromosome elimination may apply case-by-case in ultrawide hybrids, depending on the parental species involved.

Uniparental genome elimination in hybrids obtained between members of the botanical tribe of Triticeae is almost exclusively restricted to the male parent. A closer analysis of a recent literature survey on parental genome elimination (Ishii et al. 2016) revealed 65 intergeneric or interspecific hybrid combinations that involved one or both parents from within the Triticeae tribe. Of these 65 hybrid types, only four combinations (6%) were characterized with maternal genome elimination, whereas the majority (94% or 61 hybrid types) lost, partially or completely, the paternal genome. Forty-three hybrid combinations (66%) produced only haploids, i.e., completely lost the maternal (three hybrids) or the paternal (40 hybrids) genome. Another five combinations (8%) also contained (besides generating maternal haploids) the partial or full paternal genome: the only intergeneric combination of these was hexaploid wheat × barley (*T. aestivum* × *H. vulgare*), and the other four combinations being interspecific *Hordeum* hybrids again with *H. vulgare* as the male parent. All these 48 combinations (74%) required in vitro embryo rescue to generate viable plants. The remaining 17 hybrid types (26%) resulted in haploids (one paternal and the rest maternal), and full, but no partial, hybrids.

Complete genome elimination as well as its absence in ultrawide cereal hybrids are both of practical significance and desirable for genetic improvement. In the first case, the resulting maternal (doubled) haploid lines represent valuable breeding material, especially in hybrid maize programs (Weber 2014). Partial or full hybrids, on the other hand, provide useful pre-breeding lines for the sexual, non-GM transfer of desirable, agronomic or disease resistance, traits. It is, therefore, important, for both scenarios, to collect reliable information about the distribution and dynamics of the parental chromosomes in interspecific cereal hybrids. As highlighted above, the only intergeneric combination that has produced the full range of paternal chromosome series is hexaploid wheat (♀, *n* = 21) × barley (♂, *n* = 7), comprising maternal haploids (zero barley chromosomes) and partial (1–6 chr.) as well as full hybrids (7 chr.). Therefore, this combination was selected to study the frequency of chromosome elimination and stability of individual chromosomes in ultrawide hybrids.

As expected for paternal chromosome elimination, a high frequency of maternal haploids is often reported in wheat × barley hybrids. In four independent studies, the (relative) frequencies of wheat haploids were as follows: nine haploids in ten hybrid plants (90%) from two cross combinations (Finch and Bennett 1982), 19/35 (54%) from eight combinations (Taketa et al. 1995), 50/78 (64%) in five combinations (Taketa and Takeda 1997), and 32/42 (76%) in three wheat × one barley genotype combinations (Polgári et al. 2014). Since there was not a single overlap among the 18 cross combinations tested in total, the complete elimination of the barley genome in wheat maternal background appears to be a general phenomenon and not restricted to specific genotype combinations.

Recently, we reported on an efficient hybridization system between hexaploid wheat and barley (Polgári et al. 2014), the two most important cereals in the temperate zone. Here, we have made use of this system, and generated a high number of partial or full hybrid plants to make a thorough inventory of barley chromosome distribution in the population and to enable a statistically meaningful analysis on the mode (random or preferential) of chromosome elimination. For this purpose, 210 plants derived from two wheat × barley cross combinations were analyzed by barley chromosome-specific markers and the frequency of eliminated/retained chromosomes was calculated. Statistical analysis of the data indicates no preferential elimination and, thus, a random distribution of barley chromosomes in wheat background during the early vegetative (post-zygotic) development.

## Materials and methods

### Plant material

The primary female parent was ‘Sichuan’, a Chinese hexaploid spring wheat (*T. aestivum* L.; 2*n* = 6*x* = 42, AABBDD genomes) described by Polgári et al. (2014). A secondary female parent (in one-third of the crosses) was CS *Ph*^*I*^ (line C04-1), a ‘Chinese Spring’ (CS) derivative that contains the *Ph*^*I*^ gene from *Aegilops speltoides* Tausch, an epistatic suppressor of the *Ph1* (pairing homoeologous) gene in wheat (Chen et al. 1994; received from Friebe, Kansas State University). This line contains a small non-Robertsonian translocation (42, T3BL·3BS/3SS) between CS and *Ae. speltoides* (Li et al. 2011) to induce meiotic pairing of homoeologous chromosomes. The six-row spring barley (*H. vulgare* L.; 2*n* = 2*x* = 14, HH genome) cultivar ‘Morex’ (Rasmusson and Wilcoxson 1979) was the only male parent.

### Pollination and embryo rescue

Pollinations were carried out on potted wheat plants grown under field conditions. Spikes were emasculated by first removing the apical and basal spikelets, and then all the inner florets in the rest of the spikelets, except for the two outermost ones. In total, 106 spikes were pollinated with barley pollen. One day after pollination (DAP), the spikes were treated by injection with 100 ppm 2,4-D (2,4-dichloro-phenoxyacetic acid, Sigma-Aldrich) to promote pseudo-seed development. Germination and regeneration of embryos excised 14–16 DAP took place on modified N6 medium (Chu et al. 1975).

### DNA marker analysis

Frozen leaf pieces (10 mg) from young plants before tillering (Zadoks scale: Z13–Z14) were homogenized with two 3-mm stainless steel beads in the TissueLyser II laboratory mixer-mill disruptor (Qiagen NV, Hilden, Germany) for 2 × 1.5 min at 29 Hz. Total DNA was isolated by adding 100 µL of AquaGenomic™ solution (MoBiTec GmbH, Göttingen, Germany) and subsequent precipitation with isopropanol according to the manufacturer’s instructions. Qualitative and quantitative evaluation of the purified DNA was done with ND-1000 spectrophotometer (NanoDrop, Thermo Fisher Scientific) to meet the following criteria: peak of absorbance (A) scan at 260 nm, A260/A280 ≥ 1.8, A260/A230 ≥ 2.0.

The selected set of barley chromosome-specific STS (sequence-tagged site), SSR (simple sequence repeat), and gene-specific markers is listed and characterized in Table 1. PCR amplifications were carried out in 20 µL of reaction mixture-containing 1.0 µL of template DNA, 0.2 µL (1 U) of DreamTaq™ DNA polymerase (Fermentas, Thermo Fisher Scientific), 2.0 µL of 2 mM dNTPs, 2.0 µL of 10× DreamTaq™ buffer, and 0.5–0.5 µL of each 0.5 mM primer (Table 1). The reactions were performed in Veriti^®^ thermal cyclers (Applied Biosystems, Thermo Fisher Scientific) with the following conditions: denaturation at 98 °C for 5 min; 40 cycles at 98 °C for 5 s, X°C (depending on the marker, Table 1) for 10 s, and 72 °C for 25 s; a final extension at 72 °C for 7 min. In parallel with each hybrid sample, all the primer pairs, including the universal control primer set (UPP, Ta30797: Paolacci et al. 2009), were tested on samples from the wheat and barley parent as well as on non-template (negative) controls. Amplification products (10 µL) were separated in a 2% agarose gel, stained with ethidium bromide, and visualized in the G:Box gel documentation system (Syngene, Synoptics Ltd, UK). Two biological and at least two technical replications were performed for each marker.

**Table 1:** Characterization of the DNA markers used in this study

Chromosome arm	Gene/locus		Primer sequence (5′–3′)	*T*_m_	Product size (bp)
1HL	HvCENH3α^a^	F	AGA AGA AGA TCG GGT CCG CTA	66.4	801
		R	GTG CAA ACG GGA TGA GAA AAT T	66.2	
2HS	HvM36^b^	F	TCC AGC CGA CAA TTT CTT G	64.2	114
		R	AGT ACT CCG ACA CCA CGT CC	64.5	
3HL	ABG377^a^	F	GCT GCT ATG AGG AGA GAA CC	60.6	507
		R	TGG TAT GAA ACA GGT GAA TA	55.4	
4HL	ABG498^a^	F	TTA CTG AAG AAA AAC CTG TC	53.4	509
		R	CTG ACT ACT GGA TGG ACC AC	59.6	
5HL	HvCsIF7^a^	F	CCC TGC TCT TGC TTG TCG TAG	66.1	121
		R	TAG CCA AGC AAT TGC ATT T	60.8	
6HS	HvCENH3β^a^	F	ATG GCT CGC ACG AAG AAA ACG G	73.1	453
		R	GTC GGC TTG CTC TCC TTC TTG TTC G	73.4	
7HS	ABC465^a^	F	CAC GAC AGA CGG ACC AAA TG	66.6	438
		R	GCT ACT GGG ACA AAA TCT CC	60.1	
Control (UPP)	Wheat cDNA (TA30797)	F	GCCGTGTCCATGCCAGTG	58.1	195
		R	TTAGCCTGAACCACCAGTGC	60.1	

*UPP* universal plant primers (Paolacci et al. (2009), *F* forward, *R* reverse

^a^STS, sources: HvCENH3α and β, Sanei et al. (2011); ABG377/498 and ABC465, Kleinhofs et al. (1993); HvCsIF7, Burton et al. (2008)

^b^(GA)_13_ SSR, source: Liu et al. (1996)

### Genomic in situ hybridization (GISH)

Young roots from tillering wheat × barley hybrid plants in pots were collected before 10 am, and treated with nitrous oxide gas (10 bar, 2 h) at room temperature, as described by Kato (1999). Treated roots were then fixed in Clarke’s solution (1 part glacial acetic acid, 3 parts absolute ethanol) for the preparation of mitotic metaphase chromosomes. Squash preparations were made in 45% acetic acid and the coverslips were removed by freezing with liquid nitrogen, followed by dehydration in a graded ethanol series. The microscope slides were then air-dried overnight and stored at − 20 °C. The GISH procedure was essentially performed according to Szakács et al. (2013). Briefly, barley genomic DNA was labeled with digoxigenin-11-dUTP (Roche Diagnostics, Basel, Switzerland) by nick translation for use as a probe. Unlabeled wheat genomic DNA was sheared by autoclaving and used to block cross-hybridization at a ratio of 35:1 to the probe. Detection was done with 100× diluted rhodamine-conjugated sheep antidigoxigenin Fab fragments (Roche). The slides were counterstained with 1 µg/mL 4′,6-diamidino-2-phenylindole (DAPI) and mounted in Vectashield^®^ antifade medium (Vector Laboratories, Inc., Burlingame, USA). Hybridization signals were examined under an Axioskop-2 epifluorescence microscope (Zeiss, Oberkochen, Germany) equipped with appropriate filter sets. Images were captured with a SPOT™ CCD camera (Diagnostic Instruments, Inc., Sterling Heights, USA) and processed with the Image-Pro^®^ Plus software (Media Cybernetics, Inc., Rockville, USA) for contrast and brightness. No further manipulations were performed with the images. At least five metaphases (usually 8–12) were examined for each plant sample, which provided identical results with those of the marker analysis.

### Statistical analysis

To determine whether the null hypothesis of no significant difference between the two wheat genotypes for embryo induction and plant yield after crossing with barley can be maintained, a two-tailed two-sample *z* test as well as Chi-square test were performed for the relative frequencies obtained.

The preferential elimination of any barley chromosome was evaluated by first determining the absence or presence of DNA markers for individual barley chromosomes in all 210 plants. The binary presentation of the data (0 for absence and 1 for presence of each barley chromosome) was then analyzed by cross tabulation (contingency table). The null hypothesis was that each barley chromosome has an equal chance (and thus identical frequency) of elimination. For testing statistical bias between the Observed Count and Expected Count values, the nonparametric Chi-square goodness-of-fit test was applied. Critical sample size for Chi-square analysis was determined using MS Excel’s Goal Seek capability based on the following criteria: alpha (probability of Type I error) = 0.05, power (1–beta, probability of not making Type II error) = 0.8, *df* = (number of rows–1) × (number of columns–1).

Statistical associations between chromosomes (double combinations) for nonrandom elimination were investigated first with the Chi-square test of independence. The strength of association in significant cases was further analyzed with Cramér’s *V* test (Cramér 1946). Computing was performed using IBM SPSS Statistics Version 22.0 and the Real Statistics Resource Pack software (release 5.4, http://www.real-statistics.com).

## Results

### Generation of the wheat × barley plant population

Of the 2,553 florets pollinated in total with barley pollen, 316 embryos (12.4% of florets) were collected, with an average of 70% frequency regenerating into 218 (8.5% of florets) viable plants. The corresponding figures for the two cross combinations separately were: ‘Sichuan’—1717 florets pollinated, 268 embryos (15.6%) excised, and 199 plants (11.6%) regenerated; CS *Ph*^*I*^—48 embryos (5.7%) and 19 plants (2.3%) obtained from 836 cross-pollinated florets. These figures correspond to and confirm the previous results of Polgári et al. (2014) (Table 2), which demonstrates the reproducibility of our protocol.

**Table 2:** Embryo induction and plant yield for ‘Morex’ barley (♂) crosses of two wheat genotypes

Wheat genotype (♀)	This study				Polgári et al. (2014)			
	No. spikes	No. florets	No. embryos (%)	No. plants (%)	No. spikes	No. florets	No. embryos (%)	No. plants (%)
‘Sichuan’	67	1717	269 (15.7^a^)	199 (11.6^a^)	10	285	45 (15.8^a^)	40 (14.0^a^)
CS *Ph*^*I*^	38	836	48 (5.7)	19 (2.3)	34	810	47 (5.8)	32 (4.0)
Total	105	2553	317 (12.4)	218^b^ (8.5)	44	1095	92 (8.4)	72 (6.6)

^a^Highly significant (*p* < 0.0001) difference from CS *Ph*^*I*^ for that parameter with a two-tailed two-sample *z* test and Chi-square test

^b^The 210 plants for molecular analysis (Suppl. Table 1) were composed of 194 and 16 plants from the ‘Sichuan’ and CS *Ph*^*I*^ crosses, respectively

A two-sample *z* test was performed to determine whether there was a significant difference between the two wheat genotypes with respect to the relative frequencies of embryos and plants. The *z* statistic was highly significant (*p* < 0.001) at the 0.05 critical alpha level in all the cases (Table 2). Therefore, the null hypothesis was rejected and we concluded that the difference between the two wheat genotypes was significant and ‘Sichuan’ was superior for the tested parameters of crossability with barley (‘Morex’).

### DNA marker analysis of the wheat × barley plant population

We reasoned that this population of wheat × barley hybrids is of suitable size for analyzing the distribution and elimination frequencies of paternal barley chromosomes. Of the 218 plants regenerated, eight plants were discarded because of consistently poor DNA quality. Therefore, 210 plants (194 and 16 plants from the ‘Sichuan’ and the CS *Ph*^*I*^ cross, respectively) were characterized with established chromosome-specific STS, SSR and gene-specific markers (Table 1), one for each of all seven barley chromosomes. A detailed scoring for all 210 plants is listed in systematic order (Supplementary Table 1).

**Fig. 1:**
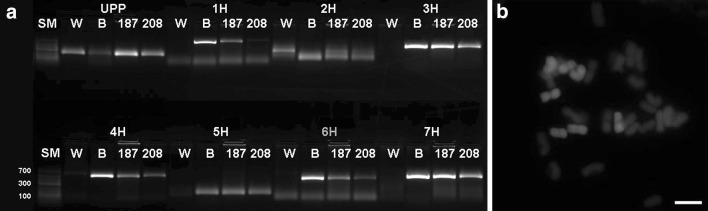
DNA marker (**a**) and cytological (**b**) characterization of wheat × barley hybrid plants. **a** Presence/absence of all chromosome-specific markers in two primary hybrid plants (Nos. 187 and 208: Supplementary Table 1) and their parents; **b** GISH analysis of the No. 187 hybrid plant containing seven barley chromosomes (*n* = 28), scale bar = 10 µm. *SM* size marker (GeneRuler™ Low Range DNA ladder, Thermo Fisher Scientific), *W* wheat (maternal) parent, *B* barley (paternal) parent, *UPP* universal plant primers (quality control)

As an illustration, the DNA marker analysis of two full hybrid plants (Nos. 187 and 208) positive for all chromosome-specific markers is summarized side by side the parental genotypes as controls (Fig. 1a). In addition, a representative screening in a panel of 45 plants for a single marker specific for the 3H chromosome (Supplementary Fig. 1) is shown with 20 negative and 25 positive individuals. To compare with the results of DNA marker screening, GISH was performed on root tips collected from tillering plants representing each ploidy group. Haploids as well as hypoploids with additional barley chromosomes—but no translocations—were identified (Supplementary Fig. 2), as expected, including the presence of full hybrids (Fig. 1b). These observations corresponded with the marker-based data obtained in the hybrid plant population (Supplementary Table 1).

### Statistical evaluation of chromosome elimination frequencies

**Fig. 2:**
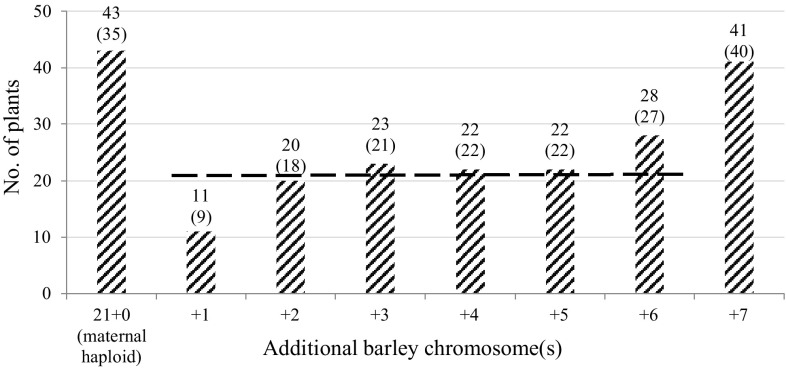
Chromosome composition of 210 plants regenerated from two wheat × barley cross combinations. The dashed line indicates the theoretical frequency (21.00) of unbiased, equal distribution for all 126 plants in the hypoploid population (Supplementary Table 2: column 7) (in brackets, data for the 194 plants of the ‘Sichuan’ × ‘Morex’ combination only)

As expected, the number of barley chromosomes in the tested 210 plants ranged between 0 and 7. A bimodal distribution of frequency was observed with statistically significant peaks for 0 (maternal haploids) and 7 (full hybrids) chromosomes (Fig. 2, Supplementary Table 2: columns 1–3 and 4–6), which represented the final and starting phases of the chromosome elimination process, respectively. After removing these two peaks, Chi-square analysis of the remaining 126 hypoploid plants (columns + 1 to + 6 on Fig. 2) indicated no significant statistical bias over all these hypoploid classes [Chisq (*df* = 5, *n* = 126) = 7.429; *p* = 0.191] (Supplementary Table 2: column 7). In other words, no preference was apparent in this population for any particular hypoploid level. This is a first indication for the random nature of chromosome elimination in this intergeneric cross combination. The same conclusion can be drawn from the statistical analysis of 119 hypoploid plants derived from the ‘Sichuan’ × ‘Morex’ combination alone ([Chisq (*df* = 5, *n* = 119) = 9.218; *p* = 0.100], Supplementary Table 2: column 8), demonstrating that the two wheat genotypes (‘Sichuan’ and CS *Ph*^*I*^) may not be different for this trait. Post hoc analysis revealed a slight underrepresentation of hypoploid class *n* + 1 (Supplementary Table 2: columns 7–10, yellow boxes), which may indicate that, once the elimination program ran down to + 1 paternal chromosome, then the remaining single chromosome will be lost more easily.

**Fig. 3:**
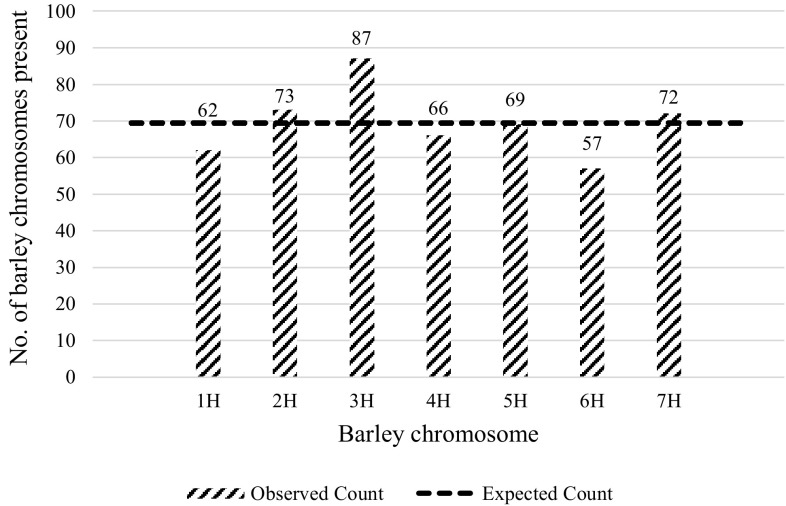
Frequencies of individual barley chromosomes (1H-7H) in 126 hypoploid (partial hybrid) plants from two wheat × barley cross combinations. The dashed line indicates the theoretical frequency (69.43) of unbiased, equal distribution for all 486 barley chromosomes in the hypoploid population (Supplementary Table 4: column 2)

After tabulation of the frequency of individual barley chromosomes separately in all hypoploid classes and in full hybrids (Supplementary Table 3), we addressed the question whether there was a preferential elimination for any individual barley chromosome in the hypoploid population (Fig. 3)? The Chi-square test for goodness-of-fit resulted in no significant outcome [Chisq (*df* = 6, *n* = 486) = 7.918; *p* = 0.244] (Supplementary Table 4: column 2), and thus, the original null hypothesis of no bias was maintained. A similar outcome was again obtained for the ‘Sichuan’ × ‘Morex’ population alone [(Chisq (*df* = 6, *n* = 468) = 7.401; *p* = 0.285), Supplementary Table 4: column 4]. In both cases, post hoc analysis indicated a weak overrepresentation of chromosome 3H (Supplementary Table 4: columns 2 and 4, yellow boxes) among all hypoploid classes. This slight effect turned out to be confined to the *n* + 1 and *n* + 2 hypoploid groups (Supplementary Table 4: columns 9–10), and, thus, did not prove to be a general, characteristic trend in the population. A comparative visual inspection of the data distributed according to various hypoploid groups (Supplementary Figs. 3–4) also confirms that this bias may have been caused by small sample sizes and restricted representations of chromosomes in groups of low hypoploid levels.

As a deeper aspect of this analysis, we also tested if there were associations in the fates of any double chromosome combination, i.e., whether such combinations occur as blocks at a higher than random frequency. We assumed that if associations in double combinations were weak or non-existent, the chance of associations among even more chromosomes would be virtually zero. Indeed, Chi-square test of independence showed no association in 13 of the possible 21 double combinations, and even in the eight remaining cases, the strength of the association was below 0.3 of V value in the Cramér’s *V* test, with five of them being weak to very weak, i.e., ≤ 0.2 of *V* (Table 3). This result confirms the random elimination of individual barley chromosomes in the analyzed wheat × barley hybrid population.

**Table 3:** Association analysis (Cramér’s *V* test) of individual barley chromosomes (1H–7H) in any double combination for absence or presence in 126 hypoploid plants obtained from two wheat × barley cross combinations

	1H	2H	3H	4H	5H	6H	7H
1H		0.292	na	na	0.193	na	0.179
2H	0.292		na	na	0.227	na	0.204
3H	na	na		na	na	na	na
4H	na	na	na		na	0.196	0.202
5H	0.193	0.227	na	na		na	na
6H	na	na	na	0.196	na		0.272
7H	0.179	0.204	na	0.202	na	0.272	

Integrated table for the pairwise comparison of each chromosome combination: figures are shown only in the cases where both Chi-square test (Supplementary Table 4) and Cramér’s *V* test were positive (*p* < 0.05). *na* no association; *V* values: 0.2–0.3 weak-moderate, ≤ 0.2 weak or very weak associations

## Discussion

Our observations are in contrast to the previous reports (Koba et al. 1991; Taketa et al. 1995), which highlighted the preferential, biased elimination of specific barley chromosomes. These authors found that barley chromosomes 1H and 5H (Koba et al. 1991) or 4H and 5H (Taketa et al. 1995) were preferentially lost in their 13 and 19 hypoploid hybrid plants, respectively (a re-analysis of the data from Taketa et al. did not reveal a statistically significant effect for any barley chromosome, Supplementary Table 4: columns 11–13). Remarkably, chromosome 4H (and 2H) was, in contrast to the conclusion of Taketa et al. (1995), the last one to get eliminated according to Koba et al. (1991). Our analysis, however, did not reveal any such preference, with the possible exception of a more stable chromosome 3H at low hypoploid levels (Supplementary Table 4, highlighted in yellow).

Besides the possible role of genotypic differences, two kinds of explanations are suggested here to account for the discrepancy between these previous studies and the present one. The first element is the size of the experiments. Koba et al. (1991) and Taketa et al. (1995) determined the chromosome constitution (by C-banding and isoenzyme markers) in 19 and 33 (not including 13 and 18 mosaic) plants, respectively, compared to 210 plants here. Thus, the size and the statistical power of these studies were too small to reliably evaluate the biased or random elimination of individual chromosomes.

The second explanation is related to the type of analysis. Chromosomes can be observed directly in dividing cells (cytologically) or identified indirectly by using genetic (DNA) markers. Cytological observation of chromosome elimination can be done in the developing proembryo and endosperm (Finch 1983; Ishii et al. 2010), or in regenerated plants as end-points. Similarly, DNA marker screening is primarily performed on plant end-products. However, the major difference between the cytological and marker approach is that the former is done on individual cells and, therefore, reveals the mosaic nature of root tip cells. Indeed, according to the data of Koba et al. (1991) and Taketa et al. (1995) the (relative) frequencies of mosaics were 13/60 plants (22%) and 18/94 plants (19%), respectively. This level of mosaicity makes precise categorization of the tested plants difficult and further weakens the power of a statistical evaluation. In contrast, DNA is extracted from a bulk of cells, and as a result, different mosaic events occurring at a low frequency are outweighed or not manifested in the consensus DNA. In addition, the exponential amplification during PCR reduces the detection of any minor mosaicism. Finally, PCR-based marker screening has a higher throughput and is much faster than direct cytological observation. For these reasons, DNA marker characterization was preferred to cytogenetical analysis in the 210 hybrid plants.

Our conclusions on the random elimination of paternal chromosomes could, to some extent, be affected by the possible presence of intergeneric translocations in the plant material. Such translocations (Friebe et al. 1996) or other chromosome and genome rearrangements (Khasdan et al. 2010) are frequently found (or induced) in interspecific and intergeneric cereal hybrids, but exclusively in subsequent, post-meiotic and amphiploid progenies of primary (F_1_) hybrids (reviewed by Jiang et al. 1994; Jones and Hegarty 2009; Khasdan et al. 2010). For instance, a quick search for Robertsonian translocations revealed some 40 cases in wheat in combination with species from ten different genera, including *Hordeum* (five cases), *Secale* (9), and *Thinopyrum* (15). All these translocations were either meiotically produced (e.g., wheat–barley: Danilova et al. 2018; Türkösi et al. 2018) or spontaneously generated and discovered in advanced progenies (Thomas et al. 1998; Berzonsky and Francki 1999). Since the developmental stage (Zadoks scale: Z13–14 or 3–4 leaf stage) of our primary plant material was deliberately chosen to set it well before the first meiosis, only the possibility of somatic translocations might still left to be considered. Spontaneous mitotic translocations have hardly ever been described in higher plants including interspecific hybrids (Wilkinson et al. 1995), but again in subsequent, post-meiotic progenies (Pašakinskienė et al. 1997; Fu et al. 2010; Tang et al. 2012). In addition, the previous cytological analyses in a total of 154 regenerated plants from wheat × barley crosses (Koba et al. 1991; Taketa et al. 1995, and our tests) as well as in primary plants of many other interspecific or intergeneric crosses (see references below) did not identify conspicuous (such as Robertsonian) translocations in somatic cells. Furthermore, parental chromosome complements in primary cereal hybrids are known to be spatially separated in the interphase (Schwarzacher et al. 1989; Leitch et al. 1990; Gernand et al. 2005) and/or during mitoses (Finch et al. 1981; Linde-Laursen and Jensen 1984; Leitch et al. 1991; Mochida et al. 2004), similar to human cells (Hua and Mikawa 2018; Reichmann et al. 2018). Finally, the uniparental elimination of chromosomes is usually completed in the early embryo during a few initial cell divisions within a time window of 5–8 DAP (days after pollination) in *H. vulgare* × *H. bulbosum* (Subrahmanyam and Kasha 1973; Bennett et al. 1976; Gernand et al. 2006), 3–4 DAP in wheat × maize (Laurie and Bennett 1989) and 1–2 DAP in wheat × *Imperata cylindrica* crosses (Komeda et al. 2007). This spatial and temporal limitation further diminishes the chance of mitotic chromosome translocations between the parental genomes. Thus, the probability of substantial numbers of intergeneric translocations that could influence the statistical outcome of our data in the post-zygotic stage of hybrid plants should be negligible.

As a consequence of their random nature of elimination in intergeneric wheat × barley hybrids, all paternal barley chromosomes may, theoretically, have a similar chance for meiotic recombination with wheat chromosomes during later plant development and in subsequent generations. It should, however, be stressed that the observations in this work are associated with purely mitotic events, and a subsequent meiosis can significantly distort in the progeny the apparently random nature of chromosome elimination by, e.g., differential chromosome pairing in a hybrid background, the effect of lethality genes as on chromosome arm 1HL (Islam and Shepherd 2000; Taketa et al. 2002) and/or nucleo-cytoplasmic interactions (Pašakinskienė et al. 1997; Gill and Friebe 2013). Thus, the results obtained here represent a baseline for further systematic studies on the characterization of chromosome elimination in ultrawide hybrids.

## Conclusion

In summary, we have generated a large population of primary plants from two wheat (♀) × barley (♂) cross combinations, and analyzed in 210 plants by DNA markers as well as statistically the frequency and pattern of paternal chromosome elimination by mitosis during early embryogenesis and vegetative development. To our knowledge, this is the first systematic and large-scale analysis on uniparental chromosome elimination in intergeneric hybrids of cereals or any plant. The analysis of our data suggests no preference in the elimination of individual barley chromosomes in wheat background. Thus, the genome elimination process—in this intergeneric combination of species, at least—can initially be considered as random. For the characterization of subsequent meiotic elimination in the reproductive phase of ultrawide hybrids, detailed investigations are essential.

### Author contribution statement

DP performed crossings, embryo rescue, and the DNA marker analysis, EM carried out the cytogenetic investigation and statistical analysis, LS designed and coordinated the study, participated in crossings, embryo rescue, and statistical analysis, and wrote the paper. All authors read and approved the manuscript for publication.

## Electronic supplementary material

Below is the link to the electronic supplementary material.

Supplementary Table 1: Detailed characterization for the absence or presence of each barley chromosome (1H-7H) in 210 plants from two wheat × barley cross combinations (DOCX 34 KB)

Supplementary Table 2: Statistical analysis of different classes of hypoploids (from zero to seven barley chromosomes) in a population from two wheat × barley cross combinations (XLSX 20 KB)

Supplementary Table 3: Distribution of individual barley chromosomes (1H-7H) in all hypoploid (and full hybrid) classes in a population from two wheat × barley cross combinations (DOCX 13 KB)

Supplementary Table 4: Statistical analysis on the persistence of individual barley chromosomes (1H-7H) in a population from two wheat × barley cross combinations (XLSX 17 KB)

Supplementary Figure 1: Representative screening for the absence (20) or presence (25) of the 3H barley chromosome (STS marker ABG 377, Table 1) in 45 wheat × barley hybrid plants (for identification, Supplementary Table 1) (DOCX 414 KB)

Supplementary Figure 2: Representative examples of various cases of chromosome elimination in wheat × barley hybrid plants as characterized by GISH (DOCX 1782 KB)

Supplementary Figure 3: Distribution (A) and relative frequency (B) of individual barley chromosomes (1H-7H) in all hypoploid groups (maternal haploid plus 1 to 6 chromosome additions) in a population from two wheat × barley cross combinations (DOCX 29 KB)

Supplementary Figure 4: Occurrence (A) and relative frequency (B) of individual barley chromosomes (1H-7H) among all hypoploid groups (maternal haploid plus 1 to 6 chromosome additions) in a population from two wheat × barley cross combinations (DOCX 3048 KB)
