# Supplementary material for: Composition and random elimination of paternal chromosomes in a large population of wheat × barley (Triticum aestivum L. × Hordeum vulgare L.) hybrids
Source: Plant Cell Rep. 2019 Apr 6;38(6):767–75. doi: 10.1007/s00299-019-02405-1 (PMC6531609; doi:10.1007/s00299-019-02405-1)
Supplement: Supplementary file 1 — Supplementary Table 1: Detailed characterization for the absence or presence of each barley chromosome (1H-7H) in 210 plants from two wheat × barley cross combinations (DOCX 34 KB) [file 299_2019_2405_MOESM1_ESM.docx]

Supplementary Table 1: Detailed characterization for the absence or presence of each barley chromosome (1H-7H) in 210 plants from two wheat **×** barley cross combinations

|  |  |  |  |  |  |  |  |  |  |
| --- | --- | --- | --- | --- | --- | --- | --- | --- | --- |
| No. | Plant ID | 1H | 2H | 3H | 4H | 5H | 6H | 7H | No. barley chr. |
| 1 | 14/1 | - | - | - | - | - | - | - | 0 |
| 2 | 14/2 | - | - | - | - | - | - | - | 0 |
| 3 | 20/2 | - | - | - | - | - | - | - | 0 |
| 4 | 20/7 | - | - | - | - | - | - | - | 0 |
| 5 | 32/3 | - | - | - | - | - | - | - | 0 |
| 6 | 34/3 | - | - | - | - | - | - | - | 0 |
| 7 | 37/7 | - | - | - | - | - | - | - | 0 |
| 8 | 37/11 | - | - | - | - | - | - | - | 0 |
| 9 | 38/3 | - | - | - | - | - | - | - | 0 |
| 10 | 38/5 | - | - | - | - | - | - | - | 0 |
| 11 | 40/4 | - | - | - | - | - | - | - | 0 |
| 12 | 109/1 | - | - | - | - | - | - | - | 0 |
| 13 | 109/3 | - | - | - | - | - | - | - | 0 |
| 14 | 109/4 | - | - | - | - | - | - | - | 0 |
| 15 | 112/1 | - | - | - | - | - | - | - | 0 |
| 16 | 116/2 | - | - | - | - | - | - | - | 0 |
| 17 | 116/3 | - | - | - | - | - | - | - | 0 |
| 18 | 117/4 | - | - | - | - | - | - | - | 0 |
| 19 | 120/2 | - | - | - | - | - | - | - | 0 |
| 20 | 204/1* | - | - | - | - | - | - | - | 0 |
| 21 | 204/2* | - | - | - | - | - | - | - | 0 |
| 22 | 210/1/1* | - | - | - | - | - | - | - | 0 |
| 23 | 210/1/2* | - | - | - | - | - | - | - | 0 |
| 24 | 228/1* | - | - | - | - | - | - | - | 0 |
| 25 | 232/2* | - | - | - | - | - | - | - | 0 |
| 26 | 235/1* | - | - | - | - | - | - | - | 0 |
| 27 | 235/4* | - | - | - | - | - | - | - | 0 |
| 28 | 1X/7 | - | - | - | - | - | - | - | 0 |
| 29 | 3X/12 | - | - | - | - | - | - | - | 0 |
| 30 | 7X/1 | - | - | - | - | - | - | - | 0 |
| 31 | 7X/2 | - | - | - | - | - | - | - | 0 |
| 32 | 7X/3 | - | - | - | - | - | - | - | 0 |
| 33 | 7X/4 | - | - | - | - | - | - | - | 0 |
| 34 | 7X/5 | - | - | - | - | - | - | - | 0 |
| 35 | 9X/4 | - | - | - | - | - | - | - | 0 |
| 36 | 9X/5 | - | - | - | - | - | - | - | 0 |
| 37 | 12X/1 | - | - | - | - | - | - | - | 0 |
| 38 | 17X/1 | - | - | - | - | - | - | - | 0 |
| 39 | 17X/2 | - | - | - | - | - | - | - | 0 |
| 40 | 21X/1 | - | - | - | - | - | - | - | 0 |
| 41 | 21X/2 | - | - | - | - | - | - | - | 0 |
| 42 | 22X/1 | - | - | - | - | - | - | - | 0 |
| 43 | 22X/2 | - | - | - | - | - | - | - | 0 43 |
| 44 | 40/2 | - | - | + | - | - | - | - | 1 |
| 45 | 114/16 | - | - | + | - | - | - | - | 1 |
| 46 | 117/6 | - | - | + | - | - | - | - | 1 |
| 47 | 3X/10 | - | - | + | - | - | - | - | 1 |
| 48 | 38/2 | - | - | - | + | - | - | - | 1 |
| 49 | 115/6 | - | - | - | + | - | - | - | 1 |
| 50 | 8X/1 | - | - | - | + | - | - | - | 1 |
| 51 | 201/1* | - | - | - | - | + | - | - | 1 |
| 52 | 201/2* | - | - | - | - | + | - | - | 1 |
| 53 | 28/1 | - | - | - | - | - | - | + | 1 |
| 54 | 39/2 | - | - | - | - | - | - | + | 1 11 |
| 55 | 32/1 | + | + | - | - | - | - | - | 2 |
| 56 | 8/1 | + | - | + | - | - | - | - | 2 |
| 57 | 109/5 | + | - | + | - | - | - | - | 2 |
| 58 | 2X/4 | + | - | - | - | + | - | - | 2 |
| 59 | 119/2 | - | + | - | - | + | - | - | 2 |
| 60 | 119/5 | - | + | - | - | + | - | - | 2 |
| 61 | 40/3 | - | - | + | + | - | - | - | 2 |
| 62 | 239/1* | - | - | + | + | - | - | - | 2 |
| 63 | 236/3* | - | - | + | - | + | - | - | 2 |
| 64 | 1X/2 | - | - | + | - | + | - | - | 2 |
| 65 | 31/1 | - | - | + | - | - | + | - | 2 |
| 66 | 114/11 | - | - | + | - | - | + | - | 2 |
| 67 | 119/6 | - | - | + | - | - | + | - | 2 |
| 68 | 20/5 | - | - | + | - | - | - | + | 2 |
| 69 | 116/1 | - | - | + | - | - | - | + | 2 |
| 70 | 9X/3 | - | - | + | - | - | - | + | 2 |
| 71 | 34/2 | - | - | - | + | - | + | - | 2 |
| 72 | 36/5 | - | - | - | + | - | + | - | 2 |
| 73 | 116/4 | - | - | - | + | - | + | - | 2 |
| 74 | 9X/1 | - | - | - | - | + | + | - | 2 20 |
| 75 | 19/2 | + | + | - | - | + | - | - | 3 |
| 76 | 20/1 | + | + | - | - | + | - | - | 3 |
| 77 | 36/2 | + | + | - | - | - | - | + | 3 |
| 78 | 3X/1 | + | - | + | - | + | - | - | 3 |
| 79 | 35/2 | + | - | + | - | - | - | + | 3 |
| 80 | 37/4 | + | - | + | - | - | - | + | 3 |
| 81 | 27/3 | - | + | + | + | - | - | - | 3 |
| 82 | 113/3 | - | + | + | + | - | - | - | 3 |
| 83 | 120/3 | - | + | + | + | - | - | - | 3 |
| 84 | 117/1 | - | + | + | - | + | - | - | 3 |
| 85 | 117/2 | - | + | + | - | + | - | - | 3 |
| 86 | 119/1 | - | + | + | - | + | - | - | 3 |
| 87 | 3X/9 | - | + | - | + | + | - | - | 3 |
| 88 | 115/2 | - | + | - | - | + | - | + | 3 |
| 89 | 37/1 | - | + | - | - | - | + | + | 3 |
| 90 | 114/12 | - | - | + | + | - | + | - | 3 |
| 91 | 37/6 | - | - | + | + | - | - | + | 3 |
| 92 | 120/4 | - | - | + | + | - | - | + | 3 |
| 93 | 232/1* | - | - | + | - | + | + | - | 3 |
| 94 | 1X/11 | - | - | + | - | + | + | - | 3 |
| 95 | 36/4/1 | - | - | - | + | + | + | - | 3 |
| 96 | 236/2* | - | - | - | + | + | - | + | 3 |
| 97 | 120/1 | - | - | - | + | - | + | + | 3 23 |
| 98 | 115/3 | + | + | + | + | - | - | - | 4 |
| 99 | 10/1 | + | + | + | - | + | - | - | 4 |
| 100 | 23/2 | + | + | + | - | + | - | - | 4 |
| 101 | 32/5 | + | + | + | - | + | - | - | 4 |
| 102 | 2X/1 | + | + | + | - | + | - | - | 4 |
| 103 | 20/4 | + | + | + | - | - | - | + | 4 |
| 104 | 25/2 | + | + | + | - | - | - | + | 4 |
| 105 | 37/3 | + | + | - | - | + | - | + | 4 |
| 106 | 3X/3 | + | - | + | + | + | - | - | 4 |
| 107 | 28/2 | + | - | + | - | + | - | + | 4 |
| 108 | 3X/4 | + | - | + | - | + | - | + | 4 |
| 109 | 23/1 | + | - | + | - | - | + | + | 4 |
| 110 | 32/6 | + | - | + | - | - | + | + | 4 |
| 111 | 17X/3 | + | - | - | + | + | - | + | 4 |
| 112 | 120/5 | - | + | + | + | - | - | + | 4 |
| 113 | 20/3 | - | + | + | - | + | - | + | 4 |
| 114 | 35/5 | - | + | + | - | - | + | + | 4 |
| 115 | 119/4 | - | + | + | - | - | + | + | 4 |
| 116 | 39/4 | - | + | - | + | + | - | + | 4 |
| 117 | 38/4 | - | + | - | + | - | + | + | 4 |
| 118 | 117/3 | - | + | - | - | + | + | + | 4 |
| 119 | 114/15 | - | - | - | + | + | + | + | 4 22 |
| 120 | 114/1 | + | + | + | + | + | - | - | 5 |
| 121 | 114/6 | + | + | + | + | + | - | - | 5 |
| 122 | 3X/2 | + | + | + | + | + | - | - | 5 |
| 123 | 116/6 | + | + | + | + | - | - | + | 5 |
| 124 | 114/2 | + | + | + | - | + | + | - | 5 |
| 125 | 1X/4 | + | + | + | - | + | + | - | 5 |
| 126 | 32/2 | + | + | + | - | - | + | + | 5 |
| 127 | 1X/3 | + | + | + | - | - | + | + | 5 |
| 128 | 25/1 | + | + | - | + | + | + | - | 5 |
| 129 | 117/5 | + | + | - | + | + | + | - | 5 |
| 130 | 27/1 | + | + | - | - | + | + | + | 5 |
| 131 | 24/1 | + | - | + | + | + | - | + | 5 |
| 132 | 114/4 | + | - | + | + | + | - | + | 5 |
| 133 | 2X/2 | + | - | + | + | + | - | + | 5 |
| 134 | 115/1 | + | - | + | + | - | + | + | 5 |
| 135 | 35/6 | - | + | + | + | + | - | + | 5 |
| 136 | 39/3 | - | + | + | + | + | - | + | 5 |
| 137 | 27/2 | - | + | + | + | - | + | + | 5 |
| 138 | 115/4 | - | + | + | + | - | + | + | 5 |
| 139 | 119/3 | - | + | + | + | - | + | + | 5 |
| 140 | 28/3 | - | - | + | + | + | + | + | 5 |
| 141 | 113/2 | - | - | + | + | + | + | + | 5 22 |
| 142 | 114/7 | + | + | + | + | + | + | - | 6 |
| 143 | 36/1 | + | + | + | + | + | - | + | 6 |
| 144 | 114/8 | + | + | + | + | + | - | + | 6 |
| 145 | 116/5 | + | + | + | + | + | - | + | 6 |
| 146 | 2X/6 | + | + | + | + | + | - | + | 6 |
| 147 | 36/3 | + | + | + | + | - | + | + | 6 |
| 148 | 40/1 | + | + | + | + | - | + | + | 6 |
| 149 | 114/5 | + | + | + | + | - | + | \| + \| \| --- \| | 6 |
| 150 | 236/1* | + | + | + | + | - | + | + | 6 |
| 151 | 1X/9 | + | + | + | + | - | + | + | 6 |
| 152 | 2X/8 | + | + | + | + | - | + | + | 6 |
| 153 | 3X/7 | + | + | + | + | - | + | + | 6 |
| 154 | 17/1 | + | + | + | - | + | + | + | 6 |
| 155 | 35/4 | + | + | + | - | + | + | + | 6 |
| 156 | 1X/1 | + | + | + | - | + | + | + | 6 |
| 157 | 2X/5 | + | + | + | - | + | + | + | 6 |
| 158 | 17/2 | + | + | - | + | + | + | + | 6 |
| 159 | 34/1 | + | + | - | + | + | + | + | 6 |
| 160 | 39/5 | + | + | - | + | + | + | + | 6 |
| 161 | 120/7 | + | + | - | + | + | + | + | 6 |
| 162 | 2X/7 | + | + | - | + | + | + | + | 6 |
| 163 | 9X/2 | + | + | - | + | + | + | + | 6 |
| 164 | 2X/3 | + | - | + | + | + | + | + | 6 |
| 165 | 36/4/2 | - | + | + | + | + | + | + | 6 |
| 166 | 37/8 | - | + | + | + | + | + | + | 6 |
| 167 | 114/9 | - | + | + | + | + | + | + | 6 |
| 168 | 115/5 | - | + | + | + | + | + | + | 6 |
| 169 | 117/7 | - | + | + | + | + | + | + | 6 28 |
| 170 | 2/1 | + | + | + | + | + | + | + | 7 |
| 171 | 19/1 | + | + | + | + | + | + | + | 7 |
| 172 | 20/6 | + | + | + | + | + | + | + | 7 |
| 173 | 24/2 | + | + | + | + | + | + | + | 7 |
| 174 | 25/3 | + | + | + | + | + | + | + | 7 |
| 175 | 31/2 | + | + | + | + | + | + | + | 7 |
| 176 | 32/7 | + | + | + | + | + | + | + | 7 |
| 177 | 32/8 | + | + | + | + | + | + | + | 7 |
| 178 | 34/4 | + | + | + | + | + | + | + | 7 |
| 179 | 35/1 | + | + | + | + | + | + | + | 7 |
| 180 | 35/3 | + | + | + | + | + | + | + | 7 |
| 181 | 37/2 | + | + | + | + | + | + | + | 7 |
| 182 | 37/5 | + | + | + | + | + | + | + | 7 |
| 183 | 37/9 | + | + | + | + | + | + | + | 7 |
| 184 | 37/10 | + | + | + | + | + | + | + | 7 |
| 185 | 38/1 | + | + | + | + | + | + | + | 7 |
| 186 | 39/1 | + | + | + | + | + | + | + | 7 |
| 187 | 109/2 | + | + | + | + | + | + | + | 7 |
| 188 | 112/2 | + | + | + | + | + | + | + | 7 |
| 189 | 112/3 | + | + | + | + | + | + | + | 7 |
| 190 | 113/1 | + | + | + | + | + | + | + | 7 |
| 191 | 114/3 | + | + | + | + | + | + | + | 7 |
| 192 | 114/10 | + | + | + | + | + | + | + | 7 |
| 193 | 114/13 | + | + | + | + | + | + | + | 7 |
| 194 | 114/14 | + | + | + | + | + | + | + | 7 |
| 195 | 120/6 | + | + | + | + | + | + | + | 7 |
| 196 | 120/8 | + | + | + | + | + | + | + | 7 |
| 197 | 120/9 | + | + | + | + | + | + | + | 7 |
| 198 | 229/1* | + | + | + | + | + | + | + | 7 |
| 199 | 1X/5 | + | + | + | + | + | + | + | 7 |
| 200 | 1X/6 | + | + | + | + | + | + | + | 7 |
| 201 | 1X/10 | + | + | + | + | + | + | + | 7 |
| 202 | 2X/9 | + | + | + | + | + | + | + | 7 |
| 203 | 2X/10 | + | + | + | + | + | + | + | 7 |
| 204 | 2X/11 | + | + | + | + | + | + | + | 7 |
| 205 | 2X/12 | + | + | + | + | + | + | + | 7 |
| 206 | 2X/13 | + | + | + | + | + | + | + | 7 |
| 207 | 3X/5 | + | + | + | + | + | + | + | 7 |
| 208 | 3X/6 | + | + | + | + | + | + | + | 7 |
| 209 | 3X/11 | + | + | + | + | + | + | + | 7 |
| 210 | 3X/13 | + | + | + | + | + | + | + | 7 41 |
| Frequency of each barley chromosome | | 103 | 114 | 128 | 107 | 110 | 98 | 113 | 773 (210) |

* Plants (16) obtained from the CS *Ph^I^* × barley cross
