# Supplementary material for: Composition and random elimination of paternal chromosomes in a large population of wheat × barley (Triticum aestivum L. × Hordeum vulgare L.) hybrids
Source: Plant Cell Rep. 2019 Apr 6;38(6):767–75. doi: 10.1007/s00299-019-02405-1 (PMC6531609; doi:10.1007/s00299-019-02405-1)
Supplement: Supplementary file 3 — Supplementary Table 3: Distribution of individual barley chromosomes (1H-7H) in all hypoploid (and full hybrid) classes in a population from two wheat × barley cross combinations (DOCX 13 KB) [file 299_2019_2405_MOESM3_ESM.docx]

Supplementary Table 3: Distribution of individual barley chromosomes (1H-7H) in all hypoploid (and full hybrid) classes in a population from two wheat × barley cross combinations

|  |  |  |  |  |  |  |  |  |  |
| --- | --- | --- | --- | --- | --- | --- | --- | --- | --- |
| Barley chromosome | | Number(s) added | | | | | | | Total |
| Individual | | 1 | 2 | 3 | 4 | 5 | 6 | (7) |  |
| 1H |  | 0 | 4 | 6 | 14 | 15 | 23 | (41) | 62 (103) |
| 2H |  | 0 | 3 | 12 | 15 | 16 | 27 | (41) | 73 (114) |
| 3H |  | 4 | 12 | 14 | 16 | 19 | 22 | (41) | 87 (128) |
| 4H |  | 3 | 5 | 10 | 7 | 17 | 24 | (41) | 66 (107) |
| 5H |  | 2 | 6 | 12 | 13 | 15 | 21 | (41) | 69 (110) |
| 6H |  | 0 | 7 | 6 | 7 | 13 | 24 | (41 | 57 (98) |
| 7H |  | 2 | 3 | 9 | 16 | 15 | 27 | (41) | 72 (113) |
| Total |  | 11 | 40 | 69 | 88 | 110 | 168 | (287) | 486 (773) |
