# Supplementary material for: Composition and random elimination of paternal chromosomes in a large population of wheat × barley (Triticum aestivum L. × Hordeum vulgare L.) hybrids
Source: Plant Cell Rep. 2019 Apr 6;38(6):767–75. doi: 10.1007/s00299-019-02405-1 (PMC6531609; doi:10.1007/s00299-019-02405-1)
Supplement: Supplementary file 5 — Supplementary Figure 1: Representative screening for the absence (20) or presence (25) of the 3H barley chromosome (STS marker ABG 377, Table 1) in 45 wheat × barley hybrid plants (for identification, Supplementary Table 1) (DOCX 414 KB) [file 299_2019_2405_MOESM5_ESM.docx]

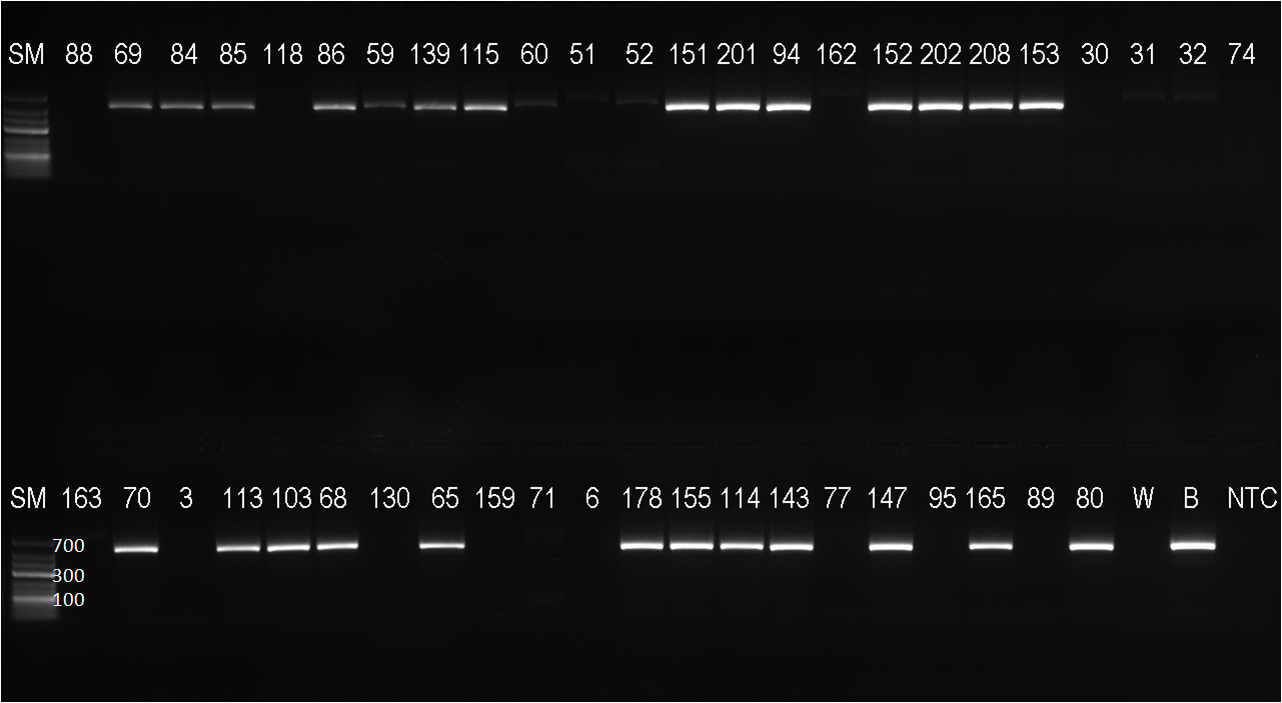


Supplementary Fig. 1: Representative screening for the absence (20) or presence (25) of the 3H barley chromosome (STS marker ABG377, Table 1) in 45 wheat **×** barley hybrid plants (for identification, Supplementary Table 1).

SM, size marker (GeneRuler^TM^ Low Range DNA ladder); W, wheat (maternal) parent; B, barley (paternal) parent; NTC, non-template control
