# Supplementary material for: Composition and random elimination of paternal chromosomes in a large population of wheat × barley (Triticum aestivum L. × Hordeum vulgare L.) hybrids
Source: Plant Cell Rep. 2019 Apr 6;38(6):767–75. doi: 10.1007/s00299-019-02405-1 (PMC6531609; doi:10.1007/s00299-019-02405-1)
Supplement: Supplementary file 6 — Supplementary Figure 2: Representative examples of various cases of chromosome elimination in wheat × barley hybrid plants as characterized by GISH (DOCX 1782 KB) [file 299_2019_2405_MOESM6_ESM.docx]

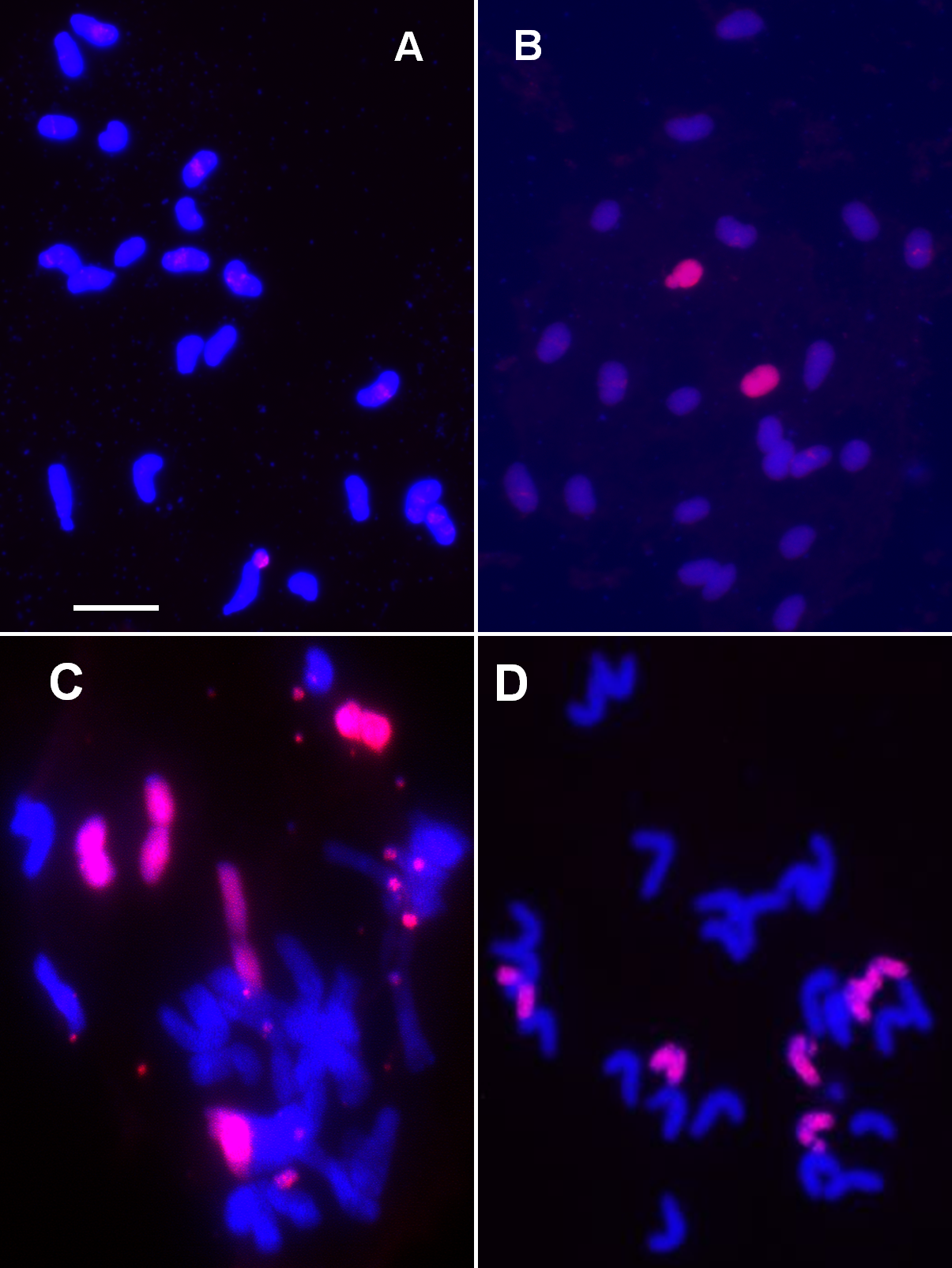


Supplementary Fig. 2: Representative examples of various cases of chromosome elimination in wheat **×** barley hybrid plants as characterized by GISH. Complete loss of all barley chromosomes results in maternal, wheat haploids (n=21, A), partial elimination leads here to the presence of two (n=23, B), five (n=26, C) and six barley chromosomes (n=27, D). Scale bar = 10 µm. A full hybrid plant karyotype (n=28) is separately shown in Fig. 2b.
