# Supplementary material for: Composition and random elimination of paternal chromosomes in a large population of wheat × barley (Triticum aestivum L. × Hordeum vulgare L.) hybrids
Source: Plant Cell Rep. 2019 Apr 6;38(6):767–75. doi: 10.1007/s00299-019-02405-1 (PMC6531609; doi:10.1007/s00299-019-02405-1)
Supplement: Supplementary file 8 — Supplementary Figure 4: Occurrence (A) and relative frequency (B) of individual barley chromosomes (1H-7H) among all hypoploid groups (maternal haploid plus 1 to 6 chromosome additions) in a population from two wheat × barley cross combinations (DOCX 3048 KB) [file 299_2019_2405_MOESM8_ESM.docx]

Supplementary Fig. 4A: Occurrence of individual barley chromosomes (1H-7H) among all hypoploid groups (maternal haploid plus 1 to 6 chromosome additions) in a population from two wheat × barley cross combinations.

Supplementary Fig. 4B: Relative frequency of individual barley chromosomes (1H-7H) among all hypoploid groups (maternal haploid plus 1 to 6 chromosome additions) in a population from two wheat × barley cross combinations.
